# Comprehensive landscape of junctional genes and their association with overall survival of patients with lung adenocarcinoma

**DOI:** 10.3389/fmolb.2024.1380384

**Published:** 2024-05-22

**Authors:** Bin Xie, Ting Wu, Duiguo Hong, Zhe Lu

**Affiliations:** ^1^ School of Information Science and Technology, Hangzhou Normal University, Hangzhou, China; ^2^ Jincheng Community Health Service Center, Hangzhou, China; ^3^ Key Laboratory of Aging and Cancer Biology of Zhejiang Province, Hangzhou Normal University, Hangzhou, China; ^4^ School of Basic Medicine, Hangzhou Normal University, Hangzhou, China

**Keywords:** lung adenocarcinoma, junctional genes, risk score, prognosis, overall survival

## Abstract

**Objectives:**

Junctional proteins are involved in tumorigenesis. Therefore, this study aimed to investigate the association between junctional genes and the prognosis of patients with lung adenocarcinoma (LUAD).

**Methods:**

Transcriptome, mutation, and clinical data were retrieved from The Cancer Genome Atlas (TCGA). “Limma” was used to screen differentially expressed genes. Moreover, Kaplan–Meier survival analysis was used to identify junctional genes associated with LUAD prognosis. The junctional gene-related risk score (JGRS) was generated based on multivariate Cox regression analysis. An overall survival (OS) prediction model combining the JGRS and clinicopathological properties was proposed using a nomogram and further validated in the Gene Expression Omnibus (GEO) LUAD cohort.

**Results:**

To our knowledge, this study is the first to demonstrate the correlation between the mRNA levels of 14 junctional genes (*CDH15, CDH17, CDH24, CLDN6, CLDN12, CLDN18, CTNND2, DSG2, ITGA2, ITGA8, ITGA11, ITGAL, ITGB4,* and *PKP3*) and clinical outcomes of patients with LUAD. The JGRS was generated based on these 14 genes, and a higher JGRS was associated with older age, higher stage levels, and lower immune scores. Thus, a prognostic prediction nomogram was proposed based on the JGRS. Internal and external validation showed the good performance of the prediction model. Mechanistically, JGRS was associated with cell proliferation and immune regulatory pathways. Mutational analysis revealed that more somatic mutations occurred in the high-JGRS group than in the low-JGRS group.

**Conclusion:**

The association between junctional genes and OS in patients with LUAD demonstrated by our “TCGA filtrating and GEO validating” model revealed a new function of junctional genes.

## 1 Introduction

Lung cancer has the highest mortality rate of all cancers. There were 2, 200, 000 incidences and 1,800,000 deaths from lung cancer in 2020 worldwide, accounting for more deaths than liver and stomach cancers combined (Leiter et al., 2023). The overall 5-year survival rate of patients with lung cancer is approximately 15%, and patients with distant metastases have an even lower survival rate of 8% (Sung et al., 2021). Most lung cancers form in the epithelial cells lining the respiratory tract. Non-small cell lung cancer (NSCLC) and small cell lung cancer (SCLC) are the two main types of lung carcinoma (Zhao et al., 2021). Lung adenocarcinoma (LUAD) is the most common type of NSCLC, accounting for more than 40% of all lung cancer cases (Denisenko et al., 2018). Moreover, approximately 70% of patients with NSCLC have inoperable local or metastatic tumors at the time of diagnosis (Katzel et al., 2009). Hence, the identification of reliable prognostic biomarkers for high-risk patients with LUAD is important for designing treatment strategies.

Most studies have identified biomarkers for diagnosis and/or prognosis. The Cancer Genome Atlas (TCGA) and Gene Expression Omnibus (GEO) databases have been widely used to predict cancer prognoses. For example, differentially methylated sites (DMSs) were selected from a TCGA-LUAD cohort, used to construct a robust DMS-based prognostic signature, and validated in a GEO cohort (Wang X. et al., 2021). Tumor mutation burden (TMB) can affect immune infiltrates and alter gene expression. A previous study stratified patients with LUAD into higher- and lower-TMB subgroups, screened nine immune genes, and used a prognostic signature based on these nine immune genes to predict patient prognoses (Zhao et al., 2021). Other studies have used similar strategies to screen biomarkers and construct LUAD prognostic nomograms based on either a group of specific genes, a gene family, or biological/physiological factors, such as lncRNA (Zeng et al., 2023), pyroptosis (Song et al., 2021), T-cell marker genes (Peng et al., 2024), tumor microenvironment-related genes (Li et al., 2023), integrin genes (Wang Y. et al., 2021; Zhang S. et al., 2023) and oxidative stress (Qian et al., 2023). Although most studies used the TCGA and/or GEO databases and incorporated the genomic profiles and clinical information to construct prediction models, some used databases such as SEER without incorporating genomic information (Zuo et al., 2021) whereas a few others were based on cohorts from local hospitals (Sun et al., 2021). Recent studies that have focused on predicting the prognosis of patients with LUAD are summarized in Supplementary Table S1. The accuracy and efficiency of some models in previous studies were compromised. Some studies only provided calibration curves, but without the area under the curve (AUC) or C-index values (Li et al., 2023; Peng et al., 2024). Similarly, others only identified independent prognostic factors but did not propose a prognostic prediction model (Zhang Z. et al., 2023). Furthermore, some studies only included a limited number of patient samples for the training and testing cohorts. External verification ensures the universality of the prognostic prediction model. However, some studies did not conduct external validation. Therefore, we aimed to develop a more accurate and reliable overall survival (OS) prediction model for patients with LUAD.

Lung cancer generally develops in cells lining air passages, predominantly originating from epithelial cells. Junctional proteins play major roles in these cells, mainly regulating cell-cell adhesion and adhering cells to the extracellular matrix. Thus, they can seal cellular sheets and control the paracellular flux of ions and solutes (Buckley and St Johnston, 2022; Kuo et al., 2022; Troyanovsky, 2023). Potential biomarkers of junctional genes have been identified to monitor diseases, including cancers (Wang D. W. et al., 2022; Hashimoto and Oshima, 2022; Parrish et al., 2022; Lin et al., 2023; Nehme et al., 2023). Altered expression of junctional genes can disrupt cell-cell adhesion, which is an initial step in cancer cell invasion. Consequently, defective cell-cell adhesion allows extra nutrients and growth factors to flow from the luminal fluid and facilitates aggressive tumor growth. During intravasation and extravasation, cancer cells disrupt cell-cell junctions and transverse through the paracellular pathways of endothelial cells that serve as a barrier for cancer cells (Wang and Liu, 2022; Wautier and Wautier, 2022). Therefore, junctional genes play important roles in tumorigenesis and cancer progression. However, the predictive value of junctional genes for the prognosis of patients with LUAD has not been studied. We used Limma and Kaplan-Meier plot analyses to screen prognosis-related junctional genes based on the genomic and clinical data from TCGA. Furthermore, we constructed a nomogram for predicting the OS of patients with LUAD and validated it in four GEO datasets.

## 2 Materials and methods

### 2.1 Retrieval of analytical data

For the TCGA-LUAD cohort, mRNA expression data and clinical information of patients with LUAD were acquired from https://portal.gdc.cancer.gov/. Originally, the data were obtained from 617 patients. Patients without overall survival data or events were subsequently excluded. We included 463 patients with LUAD and 59 healthy lung tissues as controls. The database contains 105 members of the junctional gene family, as listed in Supplementary Table S2. Four validation datasets were acquired from GEO (https://www.ncbi.nlm.nih.gov/geo/): GSE17538, GSE31210, GSE37745, and GSE72094. Each GEO dataset included 232, 226, 106, 398 patients, respectively. Among them, GSE17538 is a colon cancer cohort, which was used here as an extra external validation cohort. The rest three GSE cohorts are LUAD cohorts. Clinical information and mRNA expression data were extracted from these datasets.

### 2.2 Screening of differentially expressed genes (DEGs) using “Limma”

TCGA data comprised 24,987 genes with RNASeq data. The “Limma” package (version 3.52.4) in R (version 4.2.1, R Foundation for Statistical Computing, Vienna, Austria) was used to screen genes with differential expression in LUAD tissues compared to normal lung tissues. DEGs with |log 2-fold change (log2FC)| >1 and false discovery rate (FDR) <0.05 were considered significant, and volcano plots were constructed. Heatmaps were plotted using the “pheatmap” package in R (version 1.0.12). The “ggpubr” package (version 0.6.0) was used to draw a boxplot for observing the differential expression of the selected DEGs between LUAD and normal lung tissues.

### 2.3 Kaplan–Meier survival analysis

Kaplan-Meier plots were constructed to identify predictive DEGs for the OS of patients with LUAD. First, the “survival” (version 3.5.7) and “survminer” (version 0.4.9) packages were used to define the optimal cutoff point and draw survival curves for each low or high gene expression group. Log-rank tests were subsequently conducted to assess the predictive potential of junctional genes for the survival probability of patients with LUAD. Genes with *p* < 0.01 were considered to have significant predictive value and subjected to subsequent analyses.

### 2.4 Construction of the junctional genes-related risk score (JGRS)

Genes with *p* < 0.01 were selected from Kaplan-Meier plots and incorporated into multivariate Cox regression to develop the JGRS. For each gene, expression higher and lower than the cut-off point was designated as 1 and 0, respectively. The following formula was used to calculate the risk score of each patient with LUAD:

JGRS=∑βi×Expi

Where βi represents the coefficient of each gene and Expi represents the designated gene expression value, which was either 1 or 0. The association between JGRS and eight clinical characteristics, including sex, age, *p*-stage, tumor (T), node (N), metastasis (M) stage, and immune and stromal scores, was calculated. The “estimate” package (version 1.0.13) in R was used to obtain the immune and stromal scores. The Wilcoxon rank-sum test was used for paired comparisons, and *p* < 0.05 was considered significant. Patients were then stratified into high- and low-JGRS groups according to the JGRS cutoff point, and Kaplan-Meier curves were plotted to compare survival between the two groups.

### 2.5 Development and assessment of the nomogram

OS is the life span of patients upon pathological diagnosis until the day of death or the last follow-up. Here, patients were censored if they were alive or had no adverse events at the last follow-up visit. Nomograms are commonly employed to model cancer prognosis by combining all predictors. In the present study, age, sex, and *p*-stages were defined as continuous, binary, and multiple categorical variables, respectively. Each predictor contributed to a score, and then a final total point was obtained by summing all the contributors and scaling to the axis of the probabilities of survival to predict the 1-, 2-, and 3-year OS probabilities (Iasonos et al., 2008). The area under the curve (AUC) by “timeROC” package (version 0.4), C-index by “simplevis” package (version 7.0.0), calibration curves by “rms” package (version 6.5.0), and decision curve analysis (DCA) by “ggDCA” package (version 1.2) were used to determine the effectiveness of the nomogram. The nomogram was further validated using four GEO cohorts.

### 2.6 Gene set enrichment analysis (GSEA)

GSEA is an efficient analytical method that focuses on two opposing biological states to determine statistically significant differences in biological pathways (Subramanian et al., 2007). The “clusterProfiler” package (version 4.4.4) was used to conduct GSEA to identify functionally relevant pathways regulated by JGRS; *p* < 0.05 was considered statistically significant.

### 2.7 Analysis of the tumor immune microenvironment (TIME)

CIBERSORT and TIMER methods based on the “IOBR” R package (version 0.99.9) were used to evaluate the infiltration condition of immune cells. The results were visualized using the “ggplot2” package (version 3.4.2). Furthermore, the correlation between the expression of each gene input into the JGRS formula and abundance of immune cells were analyzed through “xCell” package (version 1.1.0) and demonstrated using “ggplot2” package (version 3.4.2).

### 2.8 Analysis of the genetic mutation status in the low- and high-JGRS groups

Somatic mutations in the TCGA-LUAD cohort, including nonsynonymous and synonymous mutations, were downloaded from https://portal.gdc.cancer.gov/. Significantly differentially mutated genes (*p* < 0.05) between the low- and high-JGRS groups were screened, and the correlations between these mutated genes were analyzed using “maftools” (version 2.14.0). Only genes mutated more than 30 times in at least one group were considered. The statistical test for the frequency of mutations was evaluated through a one-sided z-test and two-sided Chi-square test; *p* < 0.05 was considered significant.

## 3 Results

### 3.1 Clinical information of LUAD cohorts and the analytical scheme of the study

**FIGURE 1:**
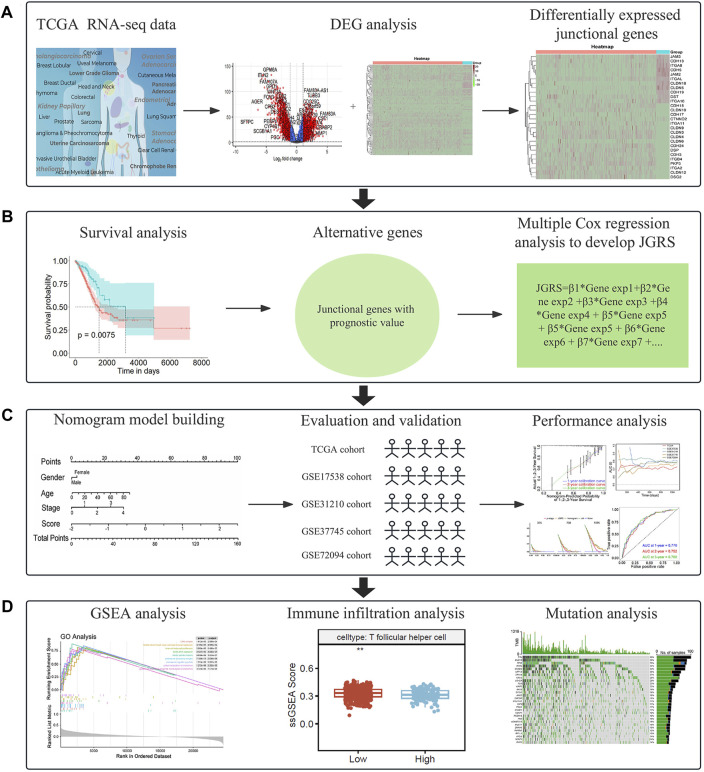
A flowchart of the study. **(A)** Screening of differentially expressed junctional genes between normal lung and LUAD tissues. **(B)** Development of a JGRS incorporating junctional genes with prognostic value. **(C)** Establishment, evaluation, and validation of the nomogram. **(D)** Functional analysis of the mechanisms behind the different JGRS.

Clinical and mRNA expression data were extracted from the TCGA database, including 59 normal and 463 LUAD samples. The median age of patients with LUAD in the TCGA cohort was 65 years, and all patients were between 33 and 88 years of age. Among them, 213 patients were men (46%) and 250 were women (54%). Information on the tumor (T), node (N), as well as metastasis (M) and *p*-stages is shown in Table 1. Clinical information of the four GEO cohorts is provided in Supplementary Table S3. We first screened differentially expressed junctional genes between normal lung and LUAD tissues. The genes associated with OS of patients were used to establish the JGRS. Subsequently, we proposed a nomogram incorporating the JGRS and corresponding clinical parameters and further validated it in four GEO cohorts. In addition, the characteristics of the biological pathways, immune infiltration, and mutational status between the low- and high-JGRS groups were analyzed. The workflow for screening the potential junctional gene prognostic panel is shown in Figure 1. DEGs, heat maps, Kaplan-Meier survival curves, nomogram interactive line diagrams, ROC curves, calibration curves, GSEA, and gene mutation status analyses were performed. The corresponding results were generated using RStudio (version 4.2.1).

**TABLE 1:** Clinical characteristics of patients with LUAD in the TCGA cohort.

Characteristics	TCGA cohort (*N* = 463)	
	N	%
Sex		
Men	213	46
Women	250	54
Age		
Mean (SD)	65.0	10.1
Median [Min, Max]	65.0	[33.0, 88.0]
Na	10	2.2
T stage		
T1	162	35
T2	243	52.5
T3	40	8.65
T4	15	3.2
TX	3	0.65
N stage		
N1	320	69.1
NX	143	30.9
M stage		
M0	304	65.7
M1	20	4.3
MX	139	30
Pathological stage		
Stage I	259	56
Stage II	108	23.3
Stage III	68	14.7
Stage IV	21	4.5
Na	7	1.5

### 3.2 Identification of DEGs

**FIGURE 2:**
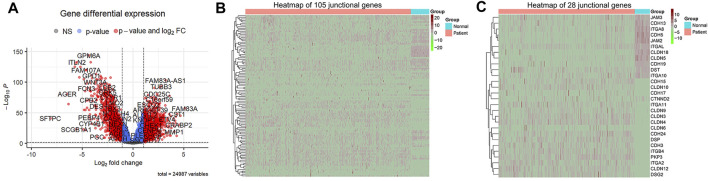
Identification of DEGs of TCGA datasets. adj. *p* < 0.05 and |log2FC| > 1 were used as the cut off criteria. **(A)** A volcano map of DEGs, which are denoted in red. **(B)** A heat map of the mRNA expression of 105 junctional genes between normal lung and LUAD tissues. Genes whose expression was upregulated are shown in red; the expression levels increase as the color darkens. Genes whose expression was downregulated are shown in green; the expression levels decrease as the color darkens. **(C)** A heat map of the mRNA expression of 24 differentially expressed junctional genes.

**FIGURE 3:**
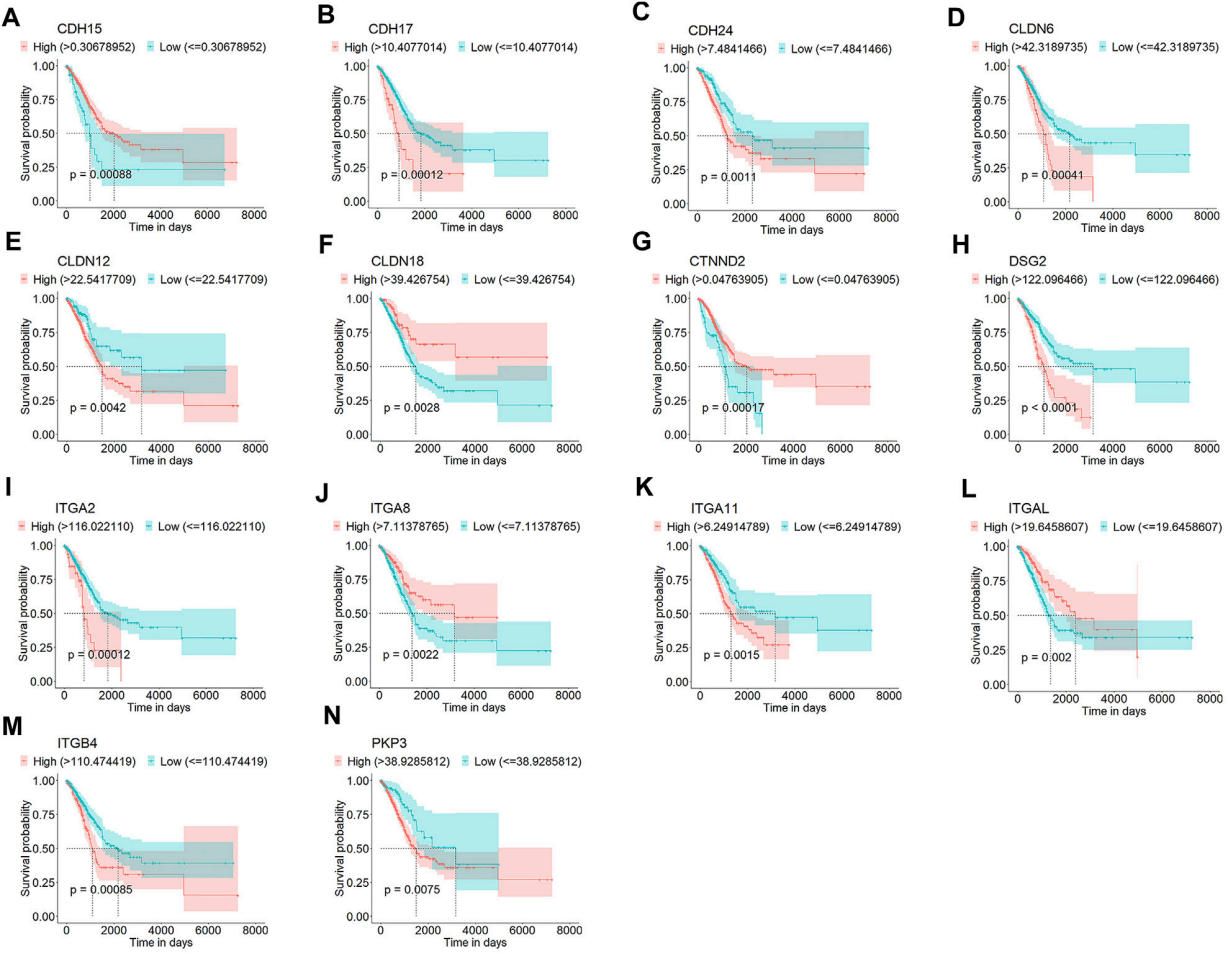
Kaplan-Meier survival curves for patients with LUAD grouped based on low and high expression levels of *CDH* *15* **(A)**, *CDH17* **(B)**, *CDH24* **(C)**, *CLDN6* **(D)**, *CLDN12* **(E)**, *CLDN18* **(F)**, *CTNND2* **(G)**, *DSG2* **(H)**, *ITGA2* **(I)**, *ITGA8* **(J)**, *ITGA11* **(K)**, *ITGAL* **(L)**, *ITGB4* **(M)**, and *PKP3* **(N)** in the TCGA cohort.

To identify the DEGs associated with OS of patients with LUAD, we first compared the mRNA expression profiles of normal lung and LUAD tissues in the TCGA cohort. Of the 24,987 genes identified, 105 were junctional genes (Supplementary Table S2). Moreover, 1716 and 1,667 genes whose expression was upregulated and downregulated, respectively, were identified; the volcano plot is shown in Figure 2A. A heat map of the expression of 105 junctional genes in normal lung and LUAD tissues was plotted (Figure 2B). Of the 3,383 genes with altered expression, 28 were junctional genes, as shown in Figure 2C. These junctional genes showed significant differences in expression between normal lung and LUAD tissues (Supplementary Figure S1). Of the 28 junctional genes identified, the expression of 17 (*CDH15, CLDN10, CDH17, CTNND2, ITGA11, CLDN9, CLDN3, CLDN4, CLDN6, CDH24, DSP, CDH3, ITGB4, PKP3, ITGA2, CLDN12,* and *DSG2*) was significantly upregulated in LUAD tissues, whereas that of 11 genes (*JAM3, CDH13, ITGA8, CDH5, JAM2, ITGAL, CLDN18, CLDN5, CDH19, DST*, and *ITGA10*) was significantly downregulated in LUAD tissues. The 28 junctional genes were then subjected to Kaplan-Meier survival analysis to screen for genes associated with the OS of patients with LUAD. Our analyses revealed 14 junctional genes, namely *CDH15, CDH17, CDH24, CLDN6, CLDN12, CLDN18, CTNND2, DSG2, ITGA2, ITGA8, ITGA11, ITGAL, ITGB4*, and *PKP3*, whose expression was significantly associated with OS (Figures 3A–N), indicating that they were valuable prognostic predictors. Additionally, we used four external databases and the samples from our local hospital to confirm the mRNA and protein expression of these 14 junctional genes. Among the four GSE validation cohorts, GSE31210 cohort contains the mRNA expression information of both LUAD tissues and normal lung tissues, so we compared the mRNA expression levels of 14 junctional genes in LUAD tissues with normal lung tissues. As shown in Supplementary Figure S2, the RNA expression changes of 14 junctional genes all coincided well with these in the TCGA cohort. Then, the clinical Proteomic Tumor Analysis Consortium (CPTAC) database was used to explore protein expression levels in LUAD tissues. The results showed that the protein expression levels of CDH15, DSG2, ITGA11, and PKP3 were significantly upregulated, whereas these of CLDN18, ITGA8, and ITGAL were significantly downregulated in LUAD tissues compared with these in normal lung tissues. The protein expression changes of the above seven junctional genes in LUAD tissues coincided well with their mRNA expression changes in the TCGA and GSE31210 cohorts. There were no significant protein expression changes of CDH17, CTNND2, ITGA2, and ITGB4 and no data of CDH24, CLDN6, and CLDN12 protein expression in the CPTAC database (Supplementary Figure S3). Furthermore, we compared the mRNA expression levels of 14 junctional genes in normal lung epithelial cells-ciliated cells with these in the lung cancer cells, using the data from The Human Protein Atlas database (HPA, www.proteinatlas.org). LUAD originates mainly from the epithelium of the bronchi, and ciliated cells are the predominate epithelial cells in the respiratory tract (Li et al., 2021). We used the average expression data of a total of 232 lung cancer cell lines available on the website. It was found that except for *CTNND2* and *ITGA2*, the mRNA expression changes of the rest 12 junctional genes all coincided well with their changes in TCGA and GSE31210 cohorts in that the mRNA expression levels of *CDH15*, *CDH17*, *CDH24*, *CLDN6*, *CLDN12*, *DSG2*, *ITGA11*, *ITGB4*, and *PKP3* were increased and the mRNA expression levels of *CLDN18*, *ITGA8*, and *ITGAL* were decreased in 232 lung cancer cell lines compared with these in ciliated cells (Supplementary Figure S4).

We also collected 9 pairs of LUAD tumor samples and their corresponding adjacent normal tissues from the affiliated hospital of Hangzhou Normal University with subjects’ informed consent and tested the mRNA expression levels of the 14 junctional genes (primers used were provided in Supplementary Table S4). As depicted in Supplementary Figure S5, consistent with the TCGA and GSE31210 cohorts, the mRNA expression levels of *CLDN12* and *ITGA11* were significantly increased and the mRNA expression levels of *CLDN18*, *CTNND2*, *ITGA8*, and *ITGAL* were significantly decreased in LUAD tissues compared with these in normal lung tissues. For *CDH15*, *CDH17*, *CLDN6*, *DSG2*, *ITGA2*, and *ITGB4*, although their mRNA expression changes did not reach to significant differences, the trends of changes were consistent with these in the TCGA and GSE31210 cohorts. It maybe because the number of our patient samples were not enough, and increasing the sample size may improve the results.

### 3.3 Development of the JGRS and its association with clinical characteristics

The prognostic risk score was computed as follows:

$$\text{JGRS}=\sum {Coef}_{i}\times {\mathrm{Exp}}_{i}$$

**FIGURE 4:**
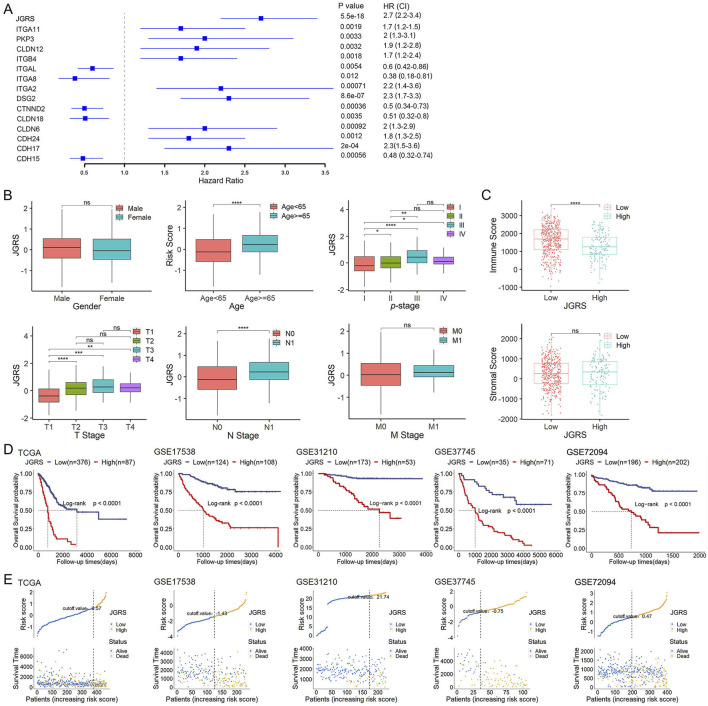
The JGRS can distinguish different clinicopathological features of LUAD. **(A)** A forest plot of the univariate Cox regression analysis of JGRS and 14 genes that were chosen for establishing a prognosis signature. **(B)** Different analyses of JGRS distribution based on sex, age, p-stage, as well as T, N, and M stages in TCGA cohort. **(C)** Distribution of immune and stromal scores between low- and high-JGRS groups in TCGA cohort. **(D)** Kaplan-Meier survival curves based on the JGRS in TCGA and four GEO cohorts. **(E)** JGRS distribution in TCGA and four GEO cohorts.

Multivariate Cox regression analysis incorporating the 14 screened junctional genes was used to generate regression coefficients. The final risk model was: JGRS = −0.39599 × *CDH15* + 0.44858 × *CDH17* + 0.20612 × *CDH24* + 0.58748 × *CLDN6* − 0.05628 × *CLDN18* − 0.46681 × *CTNND2* + 0.22880 × *DSG2* + 0.55548 × *ITGA2* − 0.51734 × *ITGA8* − 0.34780 × *ITGAL* + 0.20949 × *ITGB4* + 0.32971 × *CLDN12* + 0.14356 × *PKP3* + 0.40963 × *ITGA11*. Patients were separated into low- and high-expression groups based on the cutoff value for each of the 14 genes, and hazard ratios (HRs) were calculated. *ITGAL, ITGA8, CTNND2, CLDN18,* and *CDH15* were protective factors for LUAD survival, with HRs < 1. In contrast, *ITGA11, PKP3, CLDN12, ITGB4, ITGA2, DSG2, CLDN6, CDH24,* and *CDH17* were risk factors, with HRs >1. The JGRS had the higher HR than the individual genes (Figure 4A). We investigated the correlation between the JGRS and sex, age, TNM stage, and *p*-stage in patients with LUAD (Figure 4B). The JGRS was not correlated with sex but was significantly correlated with age. Patients with LUAD older than 65 years had significantly higher JGRS than younger patients. In addition, a higher JGRS score was significantly associated with higher T, N, and *p*-stage in patients with LUAD. From *p*-stages I to III, the JGRS increased with advancements in stage. The JGRS was significantly higher in the *p*-stage IV group than in the stage I group. However, there was no significant difference between the *p*-stage IV and II groups or between the *p*-stage IV and III groups. Similar results were observed in the T stage. The JGRSs of the T2/3/4 groups were all significantly higher than those of the T1 stage group. However, there were no significant differences between each pair of the T2/3/4 stage groups. These results can be attributed to preserved cell-cell and cell-matrix contacts at the early T stage, with these contacts lost when the tumor progressed to an advanced stage. For the N stage, the N1 stage group had significantly higher JGRS than the N0 stage group. In contrast, there was no significant difference in JGRS between the M0 and M1 groups in the M stage. This may be because any T or N stage was considered an M0 stage if it did not metastasize to a distant location. However, the loss of junctional genes occurs as the tumor grows from an early T or N stage to advanced stages.

In addition to the tumor cell-cell and cell-matrix interactions, stromal and immune cells can crosstalk with tumor cells and influence cancer growth and development. Therefore, we investigated whether the change in JGRS affects the immune and stromal scores. In this study, a higher JGRS score corresponded with a lower immune score. This negative correlation was statistically significant, whereas the stromal score was not significantly associated with JGRS (Figure 4C). We then stratified the patients in TCGA-LUAD, GSE17538, GSE31210, GSE37745, and GSE72094 cohorts into low- and high-JGRS groups based on the cutoff point in each cohort. Kaplan–Meier curves showed that patients in the low-JGRS group had significantly better OS rates than those in the high-JGRS group (Figure 4D). Figure 4E shows each of the patients on the x-axis from left to right based on their JGRS values and denotes the low-JGRS patients in blue and high-JGRS patients in yellow, analyzed by ggrisk (version 1.3). A higher percentage of deaths was observed in the high-JGRS group than in the low-JGRS group. These results indicate an association between JGRS and LUAD progression and OS in patients with LUAD.

### 3.4 Establishment and assessment of a prognostic nomogram for the OS of patients with LUAD

**FIGURE 5:**
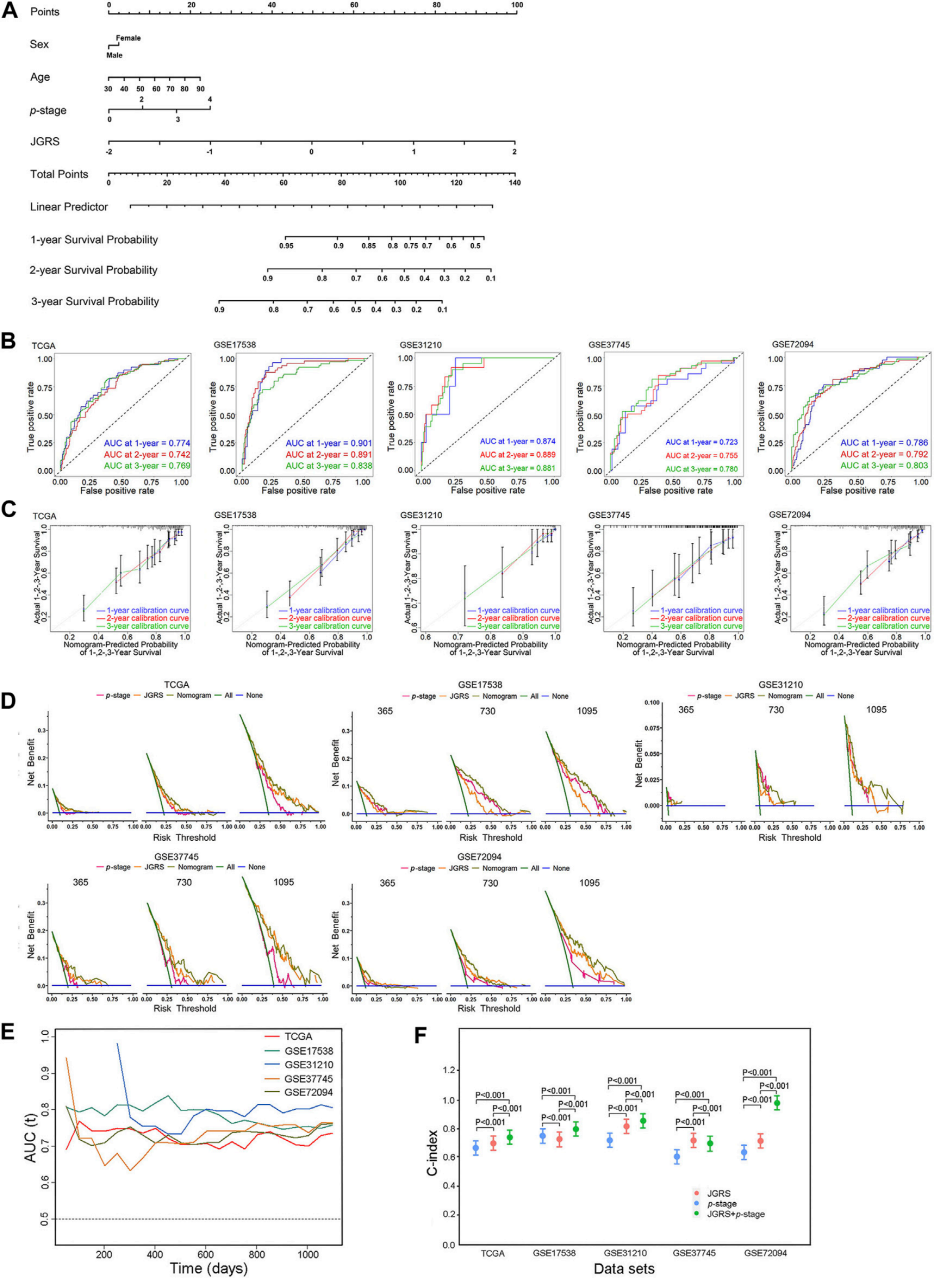
Establishment and assessment of the nomogram. **(A)** The nomogram plot was constructed based on sex, age, *p*-stage, and JGRS. ROC curves **(B)**, calibration curves **(C)**, and DCA **(D)** of the nomogram for 1-, 2-, and 3-year OS based on TCGA and four GEO cohorts. **(E)** Time-dependent AUC values in TCGA and four GEO cohorts. **(F)** C-indices of the *p*-stage, JGRS, and combined *p*-stage and JGRS in TCGA and four GEO cohorts.

According to the DEG analysis results and Kaplan-Meier plots, a nomogram was constructed to predict the 1-, 2-, and 3-year OS probabilities for patients with LUAD based on the JGRS of 14 junctional genes. As sex, age, and *p*-stage of the cancer considerably affect the OS of patients, we included these three factors in the nomogram (Figure 5A). The general performance of this nomogram was assessed using four common evaluation methods: ROC curves, calibration curves, DCA curves, and the C-index. The ROC curve depicts both the sensitivity and specificity of the regression model. The AUC of the ROC curve is an effective method for assessing the overall diagnostic accuracy of a test. As shown in Figure 5B, the AUCs for 1-, 2-, and 3-year OS in the TCGA cohort were 0.774, 0.742, and 0.769, respectively. We further validated this nomogram using four GEO cohorts: GSE17538, GSE31210, GSE37745, and GSE72094. The AUCs for 1-, 2-, and 3-year OS in the GSE17538 cohort were 0.901, 0.891, and 0.838, respectively; those in the GSE37745 cohort were 0.723, 0.755, and 0.780, respectively; those in the GSE31210 cohort were 0.874, 0.889, and 0.881, respectively; and those in the GSE72094 cohort were 0.786, 0.792, and 0.803, respectively. These results indicate that the nomogram is both sensitive and specific for predicting OS in patients with LUAD. The calibration curve shows a consensus between the predicted value of the model and the observed value. The calibration curves had good consensus in the TCGA cohort and four GEO cohorts (Figure 5C), confirming the practicality of this nomogram in predicting patient OS (Figure 5C). The DCA is a statistical method used to evaluate the clinical consequences of models and tests. The DCA for this nomogram accurately predicted the 1-, 2-, and 3-year OS rates of patients with LUAD in TCGA and four GEO cohorts (Figure 5D). Furthermore, time-dependent AUC suggested that the nomogram accurately predicted the OS of patients with LUAD, with almost all AUC values above 0.7 overtime in all five cohorts (Figure 5E). Finally, the C-index reflects the predictive ability of a model. As demonstrated in Figure 5F, *p*-stage alone produced C-indices of 0.67, 0.755, 0.724, 0.609, and 0.639 in TCGA and the four GEO cohorts, respectively. The JGRS had significantly higher C-indices than the *p*-stage in all cohorts except GSE17538, with values of 0.702, 0.733, 0.823, 0.719, and 0.717. Furthermore, C-indices of the JGRS and *p*-stage combined were 0.737, 0.799, 0.852, 0.699, and 0.982. This significantly promoted C-indices in TCGA and the GSE17538, GSE31210, and GSE72094 GEO datasets. The C-index of the combined JGRS and *p*-stage was lower than that of JGRS alone in the GSE37745 cohort. However, the C-index of JGRS remained significantly higher than that of the *p*-stage, which verified the predictive value of JGRS.

### 3.5 JGRS-related biological pathways and genes

**FIGURE 6:**
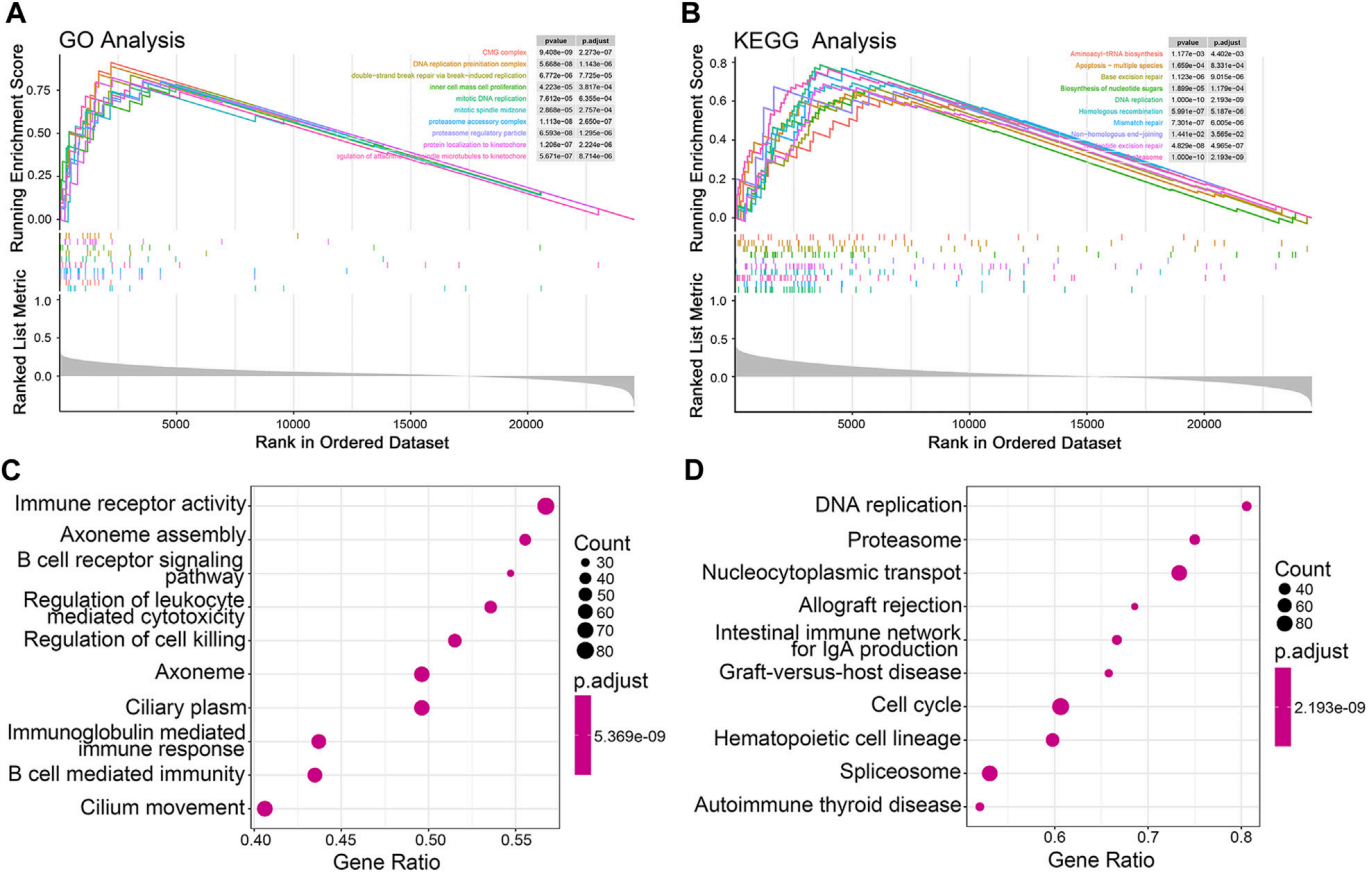
GSEA function enrichment analysis for JGRS. Curve graphs of the top 10 enriched pathways based on the GO **(A)** and KEGG **(B)** pathway databases, respectively. A dot plot of the top 10 enriched GO **(C)** and KEGG **(D)** terms, respectively.

To explore the potential mechanisms underlying the differential OS outcomes of the JGRS determination, we performed GSEA. The top 10 differentially expressed gene ontology (GO) (Figure 6A) and Kyoto Encyclopedia of Genes and Genomes (KEGG) pathways (Figure 6B) associated with the JGRS signature were identified at *p* < 0.05. Most GO and KEGG pathways enriched in the high-JGRS group were associated with cell replication (inner cell mass cell proliferation), mitotic processes (mitotic DNA replication/mitotic spindle midzone/protein localization to kinetochore/regulation of attachment of spindle microtubules to the kinetochore), and biosynthesis (aminoacyl-tRNA biosynthesis/biosynthesis of nucleotide sugars). Junctional genes primarily bind or unbind intercellular cells. Contact inhibition of proliferation occurs between cells under normal conditions, and unbinding of the cells is the precursor step of uncontrolled cell proliferation, which leads to precancerous cell development. Altered expression of junctional genes contributes to cell proliferation. For instance, high *CLDN1* expression and low *E-cadherin* expression promote cell proliferation and escape from their original sites (Bremnes et al., 2002; Wang D. W. et al., 2022). This explains the enrichment of cell proliferation and biosynthetic pathways in the high JGRS group. In addition, the top 10 GO terms for biological processes, such as immune receptor activity, B cell receptor signaling pathway, immunoglobulin-mediated immune response, and B cell-mediated immunity, were enriched in the high JGRS group (Figure 6C). KEGG enrichment analysis also revealed that immune- and cell proliferation-related categories, such as DNA replication, cell cycle, and IgA production, were enriched in the high-JGRS group (Figure 6D). This also confirmed the correlation between the immune score and JGRS (Figure 4C). In summary, the results obtained using either the GO or KEGG databases all pointed to the conclusion that the JGRS correlates with cell proliferation and immune-related processes, which may be the mechanism leading to the differences in the OS of patients with LUAD.

**FIGURE 7:**
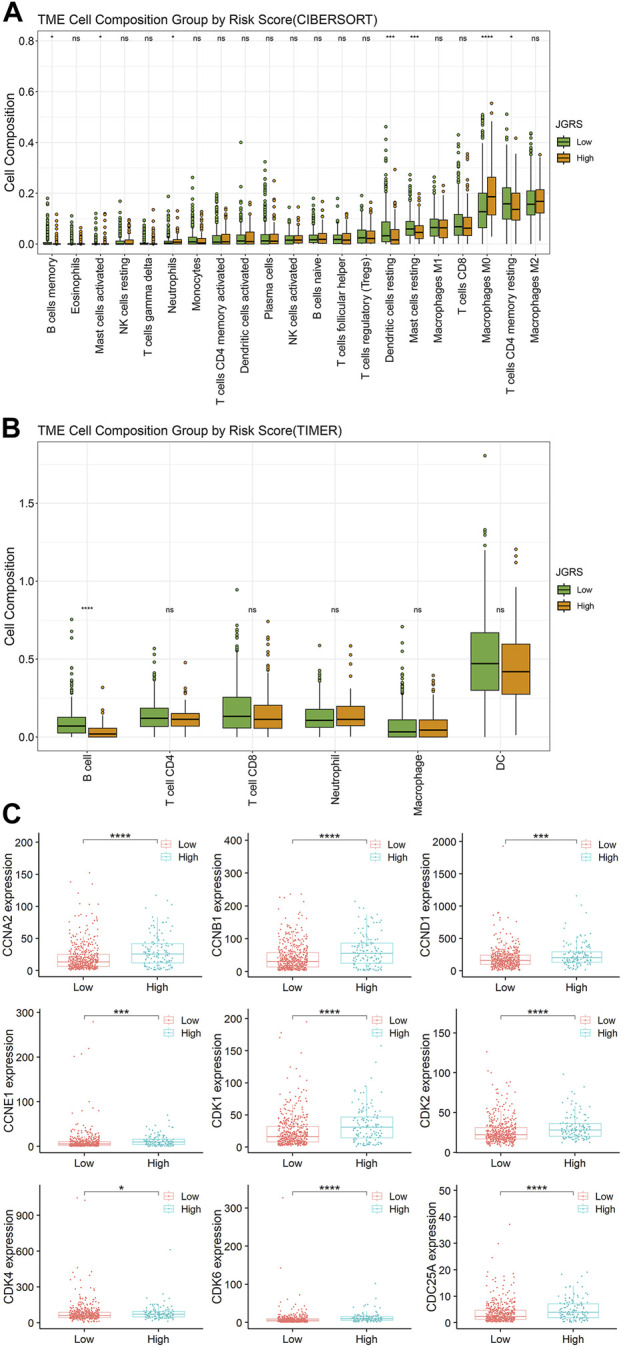
Infiltration of immune cells and the expression of cell cycle-related genes in the low- and high-JGRS groups of patients. Conditions of immune cell infiltration into the TIME based on the CIBERSORT **(A)** and TIMER **(B)** methods. **(C)** Expression of cell cycle-related genes in the low- and high-JGRS groups of patients.

An xCell analysis that included 64 different types of immune and stromal cells was conducted to further elucidate the regulatory immune cells involved in the tumor immune microenvironment (TIME). The abundance of infiltrating immune cells was determined based on the expression of marker genes. The correlations between each immune cell and the 14 JGRS signature genes are displayed as a heatmap in Supplementary Figure S6. Genes that were risk factors for OS, such as *CDH24* and *PKP3*, were negatively correlated with most immune cells, and *vice versa*. In contrast, genes that were protective factors for OS, such as *CLDN18*, *ITGA8,* and *ITGAL*, were positively correlated with most immune cells. Because the GO terms for biological processes were mainly enriched in B cell-related immunity, we investigated the infiltration of B cells into the TIME. CIBERSORT and TIMER methods were used to analyze the infiltration of different immune cell components into low- and high-JGRS tissues. As expected, both CIBERSORT (Figure 7A) and TIMER (Figure 7B) data showed a significantly higher infiltration of B cells in the low-JGRS group than in the high-JGRS group, suggesting that the poor OS rates of high-JGRS patients may be related to the reduced infiltration of B cells into the TIME. In addition to B cells, the CIBERSORT results showed a higher infiltration of dendritic cells and CD4^+^T cells in the low-JGRS group. Furthermore, the high-JGRS group was associated with cell replication, mitotic processes (Figures 6A, B), and immune regulatory features. Therefore, we analyzed the mRNA expression of cell cycle control genes. Cyclins and cyclin-dependent kinases (CDKs) are important regulators that drive cell cycle progression (Basu et al., 2022). Figure 7C shows elevated levels of cyclins A (*CCNA2*), B (*CCNB1*), D (*CCND1*), and E (*CCNE1*), as well as those of CDK1, 2, 4, 6, and Cdc25A in the high-JGRS group. These results explain the expedited cell cycle in high-JGRS patients and are consistent with the results shown in Figure 6.

### 3.6 Analysis of mutation status between the low- and the high-JGRS groups

**FIGURE 8:**
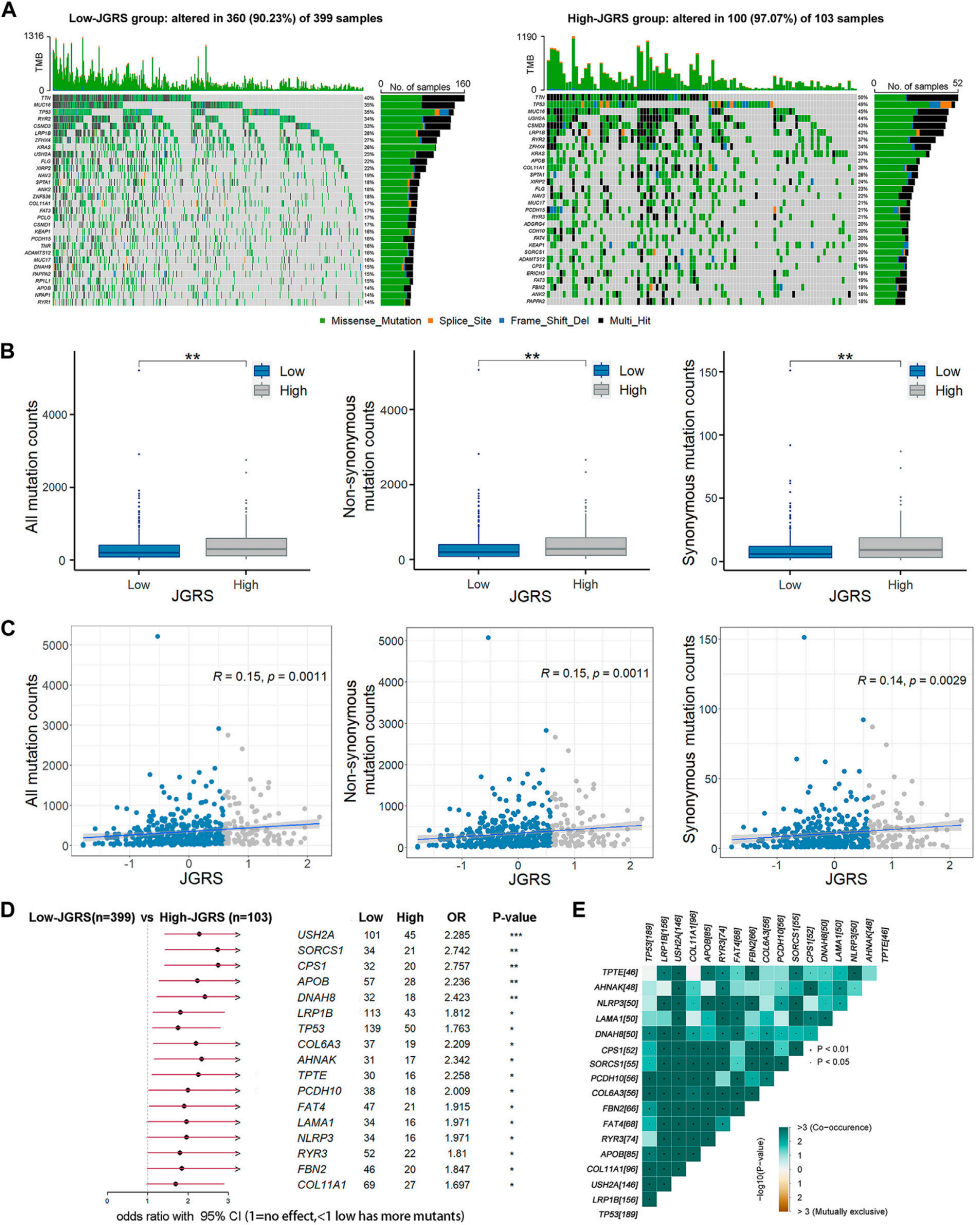
JGRS was related to tumor mutation status. **(A)** The top 30 most frequently mutated genes in the low- (left) and high-JGRS (right) groups. **(B)** The distribution of all, synonymous, and non-synonymous counts in the low- and high-JGRS groups. **(C)** Association between JGRS and all, non-synonymous, and synonymous mutation counts. **(D)** A forest plot of differentially mutated genes in the low- and high-JGRS groups, *: *p* < 0.05, **: *p* < 0.01, ***: *p* < 0.001. **(E)** Interaction effects between differentially mutated genes in the low-and the high-JGRS groups.

We analyzed the somatic mutations of patients in the TCGA cohort to further investigate the genetic mechanisms underlying the differential OS outcomes between the low- and high-JGRS groups. The 30 most frequently mutated genes in the low- and high-JGRS groups are shown in Figure 8A. The frequency of somatic mutations in the low-JGRS group was 90.23%, whereas that in the high-JGRS group was 97.07%. Further statistical analyses showed that the frequencies of total mutation counts, as well as non-synonymous and synonymous mutations, were significantly higher in the high-JGRS group than in the low-JGRS group (Figure 8B). Additionally, JGRS was significantly positively correlated with somatic, non-synonymous, and synonymous mutation counts (*p* < 0.01; Figure 8C). Comparison of the mutational frequencies of each gene revealed that 17 genes were significantly more frequently mutated in the high-JGRS group than in the low-JGRS group (*p* < 0.01), namely *USH2A, SORCS1, CPS1, APOB, DNAH8, LRP1B, TP53, COL6A3, AHNAK, TPTE, PCDH10, FAT4, LAMA1, NLRP3, RYR3, FBN2, and COL11A1* (Figure 8D). These genes were subjected to co-occurrence mutation analysis using maftools, and co-mutations were found among the 17 genes (Figure 8E).

### 3.7 Verification of prognostic DEGs using clinical tissue samples

**FIGURE 9:**
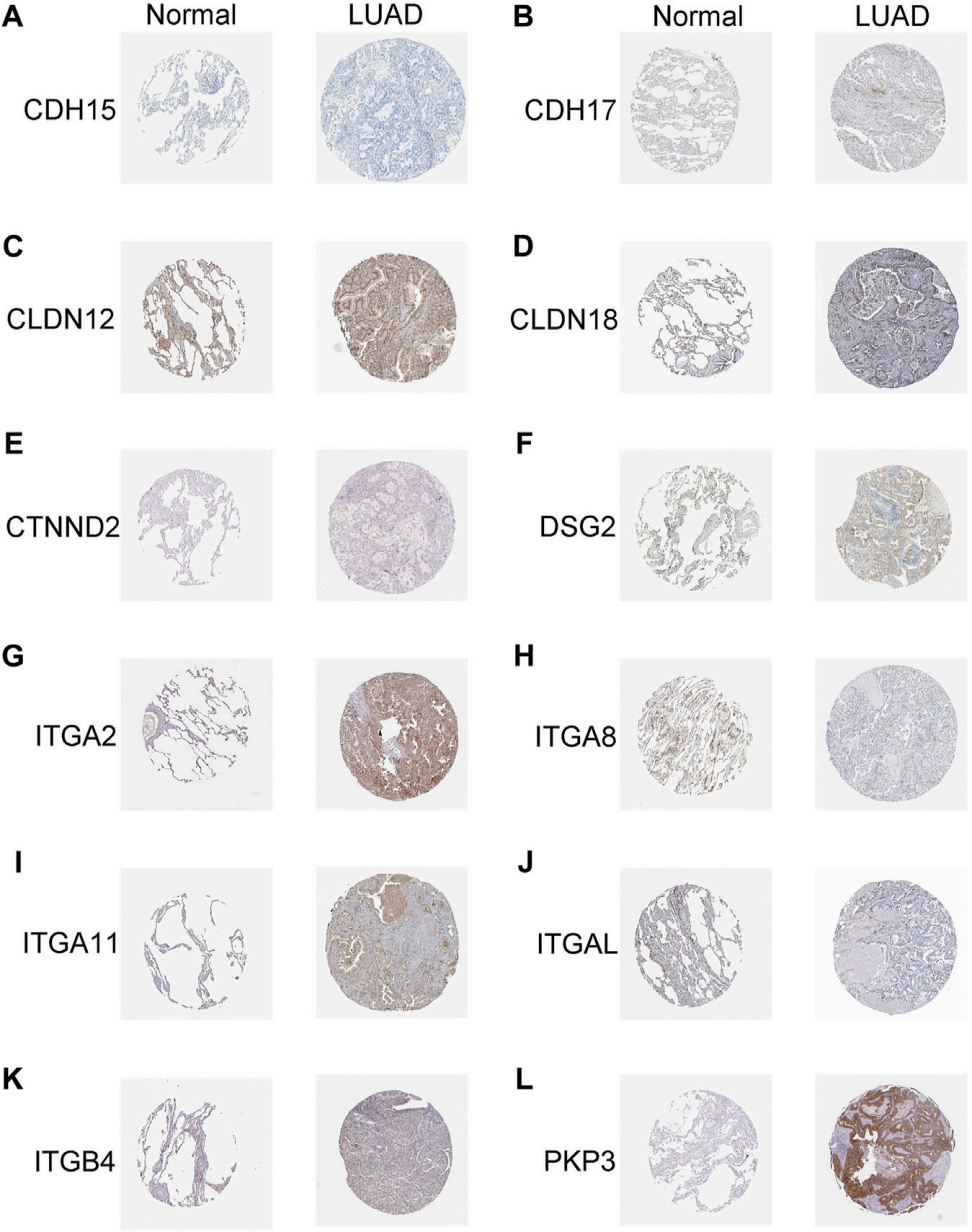
Immunohistochemical analysis of genes with prognostic values. **(A)** *CDH15*, **(B)** *CDH17*, **(C)** *CLDN12*, **(D)** *CLDN18*, **(E)** *CTNND2*, **(F)** *DSG2*, **(G)** *ITGA2*, **(H)** *ITGA8*, **(I)** *ITGA11*, **(J)** *ITGAL*, **(K)** *ITGB4*, and **(L)** *PKP3*.

To verify the reliability of the DEGs with prognostic values, we detected the protein expression of 14 genes in normal and LUAD tissues from the HPA website. Antibody-staining images except for CDH24 and CLDN6 were available on the HPA website. Of the rest 12 proteins, CDH15, CTNND2, and ITGA8, were all negatively expressed in both the normal and LUAD tissues. The expression of CDH17, CLDN12, DSG2, ITGB4, PKP3, ITGA11, and ITGA2 was all upregulated whereas the expression of ITGAL was downregulated in LUAD tissues compared to the normal lung tissues (Figure 9). These results coincide well with the JGRS formula in that *CDH17*, *CLDN12*, *DSG2*, *ITGB4*, *PKP3*, *ITGA11*, and *ITGA2* were risky factors whereas *ITGA* *L* was a protective factor for patient survival.

## 4 Discussion

Prediction of the prognosis in patients with cancer has garnered substantial research interest. Many researchers have screened for useful prognostic biomarkers using the genetic information of patients from TCGA and GEO databases or data from local hospitals combined with clinical properties to construct a nomogram that predicts patient OS. The ultimate goal was to develop an accurate and effective model. Some studies have focused on genome-wide screening by comparing cancer tissues with normal tissues, whereas others have focused on a specific gene group that targets common physiological processes or factors, such as apoptosis, cancer stem cells, tumor microenvironment, DNA methylation, gene mutation, and oxidative stress (Supplementary Table S1). In the present study, we first screened for differentially expressed junctional genes by comparing LUAD tissues with normal lung tissues. Of the 105 junctional genes identified, the expression of 28 was upregulated or downregulated in LUAD tissues. We constructed a Kaplan-Meier plot to select the genes that contributed to the OS of patients with LUAD. Fourteen of the 28 genes were selected to generate the JGRS, which was used to construct the nomogram. To the best of our knowledge, this is the first study to reveal the potential prognostic value of a panel of junctional genes.

The regulation of tumorigenesis is complex. Lung cancer often detected in the middle or late stages (Zhao et al., 2022). Approximately 40% of patients with lung cancer die from metastasis (Simonaggio et al., 2020). Migratory cells can escape from the primary site, invade normal tissues, travel through the lymphatic system or bloodstream, and spread to distant locations. Loss of cell connections is one of the initial hallmarks of epithelial cell migration (Prudkin et al., 2009). Junctional proteins mediate cell-cell and cell-matrix connections. Thus, we evaluated their role in OS prediction. Among the 14 junctional genes screened, *CLDN6, CLDN12*, and *CLDN18* belong to the tight junction family. In total, 27 claudins have been discovered to date (Osanai et al., 2017), with a high or low abundance of claudins described in diverse neoplastic tissues. For example, claudin-1 was decreased in pancreatic and ovarian cancers, as well as in LUAD (Osanai et al., 2017). Claudin-7 was reduced in LUAD (Lu et al., 2011), whereas claudin-3 and claudin-4 were increased in esophageal cancer (Osanai et al., 2017). Consistent with previous studies on the contributions of *CLDN6*, *CLDN12*, and *CLDN18* to LUAD, increased RNA expression of *CLDN6* and *CLDN12* in patients with LUAD increased the risk score and lowered the survival probability, whereas increased RNA expression of *CLDN18* had the opposite effect in the present study. Claudin-6 was significantly more frequent and most abundantly positive in adenocarcinoma (AC) than in squamous cell carcinoma (SCC), and was associated with poor prognosis in 164 patients with NSCLC from the University Hospital of Kuopio (Oini et al., 2022). This result was also confirmed in 196 patients with NSCLC at Uppsala University Hospital. These studies revealed distinct membrane-positive CLDN6 proteins in LUAD, with high CLDN6 expression associated with a worse prognosis (Micke et al., 2014). In addition, Kuner et al. conducted a global gene expression analysis of NSCLC subtypes and identified a striking presence of cell adhesion genes that were deregulated between SCC and AC subtypes. Among these, the expression of CLDN12 was upregulated in AC tissues compared to that in normal tissues (Kuner et al., 2009). Kuner et al. further detected the increased expression of other junctional genes, such as *DSG3, CLDN1, DSC2, CLDN3, CLDN7, CDH1,* and *CDH2* in the AC. However, these genes were not included in the nomogram developed in the present study. First, the genetic background of the patient tissues from their cohort (German Cancer Research Center) was, to some extent, different from that retrieved from TCGA. Second, although these genes were differentially expressed, they did not correlate with the OS of the patients; thus, they were not included in the nomogram. In addition, loss of *CLDN18* resulted in increased type 2 alveolar epithelial (AT2) cell proliferation and an increased frequency of LUAD in mice. Human LUAD, which originates from AT2 cells, also displayed reduced CLDN18 (Kotton, 2018). These findings indicate that *CLDN6, CLDN12*, and *CLDN18* markedly contribute to the prediction of OS in patients with LUAD.

Another large gene family that was incorporated into the nomogram was the integrin family. Of the selected 14 genes, five belonged to the integrin family. Integrins connect cells to the extracellular matrix. They comprise 18 α and 8 β subunits, different combinations of which can assemble into 24 complexes (Tvaroska et al., 2023). Smythe et al. discovered the downregulated expression of the subunits α2, α3, α6, and β4 and the upregulated expression of the β6 subunit in NSCLC (Smythe et al., 1995). Integrins are expressed in a histological and type-specific manner. For example, α3 was strongly expressed in AC but was infrequent in SCLC.α4 was solely expressed in bronchioloalveolar carcinoma (Guo et al., 2009). Furthermore, αvβ3 was associated with tumorigenesis and metastasis steps, leading to poor survival in patients with LUAD (Kariya et al., 2021). Integrin α6 expression was significantly higher in LUAD tissues and positively correlated with the grade and T stage of LUAD, leading to poorer patient survival rates (Shen et al., 2019). In addition, Navab et al. revealed significantly impeded growth and metastasis of lung cancer cells in integrin α11-deficient severe combined immunodeficient (SCID) mice compared to wild-type SCID mice. This demonstrated the ability of integrin α11 to promote the growth and metastasis of NSCLC. Our results were consistent with this finding, as increased RNA expression of integrin α11 in patients with LUAD increased the JGRS and decreased survival probability. Four other integrin subunits, namely, integrins α2, α8, αL, and β4, were identified as prognostic-related biomarkers in the present study. Integrins α2 and β4 were positively correlated with the JGRS and negatively correlated with patient OS, whereas α8 and αL were the opposite. As integrins play crucial roles, effective inhibitors targeting integrin subunits have been discovered and are widely used clinically, including in the fields of cardiovascular and inflammatory bowel diseases. However, the clinical development of integrin inhibitors in cancer remains considerably challenging (Slack et al., 2022).

In addition to the claudin and integrin families, other junctional genes such as cadherins, δ-catenin *(CTNND2), DSG2*, and *PKP3* were correlated with the prognosis of patients with LUAD. As these junctional genes play pivotal roles in lung tumorigenesis, the JGRS based on the selected 14 genes can be an independent prognostic marker that best reflects the prognostic status of patients with LUAD. To some extent, previous studies have provided evidence of the mechanisms by which these genes affect lung tumorigenesis, such as cell proliferation, migration, and metastasis. Our GSEA also showed that high JGRS levels were mainly associated with cell proliferation and immune regulatory pathways. Further analysis showed more abundant infiltration of B cells and upregulated expression of cell cycle-related genes, such as cyclins and CDKs in the high-JGRS group (Figure 7).

Oncogenes and tumor suppressor genes are important “players” in cancer formation, and their mutations or expression changes can lead to neoplastic transformation in normal cells. In the present study, patients were separated into high- and low-JGRS groups, and their mutational status was analyzed. Seventeen genes were mutated significantly more frequently in the high-JGRS group than in the low-JGRS group. Of these, *COL6A3, AHNAK, CPS1, TPTE*, and *DNAH8* had twice as many mutations in the high-JGRS group than in the low-JGRS group. The involvement of these genes in tumorigenesis has been reported. *COL6A3* produces the alpha (α) 3 (VI) chain of type VI collagen, a component of the extracellular matrix. Dysregulated expression of *COL6A3* has been observed in several cancers, including cervical cancer and pancreatic adenocarcinoma (Annapurna et al., 2021; Wang H. L. et al., 2022). *AHNAK*, which encodes the giant protein desmoyokin, was originally identified as a nucleoprotein in neuroblastoma cells (Sundararaj et al., 2021). It was later identified as a tumor suppressor that can negatively regulate cell growth through TGFβ signaling (Lee et al., 2014). Carbamoyl phosphate synthetase 1 (CPS1) is a tumor promoter that either supports pyrimidine synthesis or prevents the buildup of intratumoral ammonia (Yao et al., 2020). Transmembrane phosphatase with tensin homology (TPTE) shares significant homology with the tumor suppressor protein PTEN. DNAH8 is a member of the dynein axonemal heavy chains (DNAHs), and its variants have been associated with heavy smoking (Wain et al., 2016). Failure to clear the toxins in the respiratory tract due to a variant change in DNAH8 may cause lung cancer. However, there are limited studies on the subject, and this hypothesis requires further validation. Owing to their association with various cancers, these mutated genes may directly or indirectly affect the prognosis of patients with LUAD.

The performance evaluation of a predictive model is crucial following its construction. The effectiveness of a model can be evaluated through the C-index, which is often used to measure how well a biomarker predicts the time to an event. It can also be determined through AUCs, which plot the rate of true positives against false positives. A calibration plot can also be used to assess the agreement between predicted and observed values. However, a calibration plot lacks an assessed value. Thus, effectiveness can only be determined by looking at the closeness between the prediction and diagonal lines. In addition, DCA is a statistical method that evaluates models and tests their clinical consequences. Papers predicting the prognosis of patients with LUAD published in the recent 5 years are summarized in Supplementary Table S1. In total, 47 studies were included. We have listed the predictive parameters of these studies, as well as the AUC and C-indices of the training and validation cohorts. Overall, the predictive values of the models were compromised. Some studies lacked external validation or were only validated in one or two cohorts (Peng et al., 2024). Others only focused on a subgroup of patients with LUAD, such as patients with early-stage LUAD or those with metastasis, which is not applicable to all patients with LUAD. The model with the highest C-index value (0.89) was constructed by Huang et al. However, this study neither provided AUC values nor externally validated the model (Huang et al., 2021). A prognostic model with DNA methylation profiling showed promising AUC values of 0.846, 0.900, and 0.909 for 1-, 3-, and 5-year predictions, respectively. However, it was only validated in one GSE cohort (Ma et al., 2020). Our nomogram has the advantage of robust performance, as it was validated using four GEO datasets. The AUCs for TCGA and the four validation GEO cohorts were all >0.72. For two GEO cohorts, GSE17538 and GSE72094, the values were >0.78. AUCs for the 1-, 2-, and 3-year OS rates were the highest in the GSE17538 cohort, reaching 0.901, 0.891, and 0.838, respectively. These results indicate that our nomogram is both sensitive and specific for predicting OS in patients with LUAD. The C-indices of JGRS and *p*-stage combined were all above 0.73 except for one cohort, GSE37745. The values in the GSE31210 and GSE72094 cohorts were high, reaching 0.852 and 0.982, respectively. This validated the predictive ability of the model. Furthermore, the calibration curves in all four GEO cohorts showed favorable consensus (Figure 5C), and DCA showed good clinical practicability of the model (Figure 5D). Hence, our results represent a small breakthrough compared to those of previous studies.

## 5 Conclusion

This study demonstrated a significant correlation between altered expression of junctional genes and the OS of patients with LUAD. Here, we constructed a junctional gene-related nomogram model to predict the OS of patients with LUAD. Although our model was multidimensionally validated, it has some limitations. The prognosis of LUAD depends on various factors, such as patient psychology, smoking status, surgical performance, and response to radiotherapy or chemotherapy. These factors were not taken into consideration in this study because of partial records. Additionally, although we evaluated the JGRSs in patients with different TNM stages and mutation characteristics of patients in the TCGA cohort, we did not perform them in the four GEO cohorts, owing to the lack of TNM stage information and mutation data. Moreover, future studies are required to explore the underlying molecular mechanisms and subsequently advance potential clinical applications, as these cell junctional genes may be valuable therapeutic targets.

## Data Availability

Publicly available datasets were analyzed in this study. This data can be found here: NIH GDC Data Portal (https://portal.gdc.cancer.gov/projects/TCGA-LUAD) and GEO repository, accession numbers: GSE17538, GSE31210, GSE37745 and GSE72094.

## Supplementary material

SUPPLEMENTARY FIGURE S1 Expression of 28 differentially expressed junctional genes in normal and LUAD tissues.

SUPPLEMENTARY FIGURE S2 The RNA expression levels of 14 junctional genes in normal lung and LUAD tissues in the GSE31210 cohort.

SUPPLEMENTARY FIGURE S3 The protein expression levels of 14 junctional genes in normal lung and LUAD tissues by Clinical Proteomic Tumor Analysis Consortium.

SUPPLEMENTARY FIGURE S4 The RNA expression levels of 14 junctional genes in 232 lung cancer cell lines compared with normal human epithelial ciliated cells.

SUPPLEMENTARY FIGURE S5 Evaluation of the mRNA expression levels of 14 junctional genes between normal lung (*n* = 9) and LUAD specimens (*n* = 9) from our local hospital by PCR analysis.

SUPPLEMENTARY FIGURE S6 Correlation between the infiltration of 64 immune and stroma cell types with each signature genes in JGRS.
