# Corrigendum: Comprehensive landscape of junctional genes and their association with overall survival of patients with lung adenocarcinoma

**DOI:** 10.3389/fmolb.2025.1583736

**Published:** 2025-04-01

**Authors:** Bin Xie, Ting Wu, Duiguo Hong, Zhe Lu

**Affiliations:** ^1^ School of Information Science and Technology, Hangzhou Normal University, Hangzhou, China; ^2^ Jincheng Community Health Service Center, Hangzhou, China; ^3^ Key Laboratory of Aging and Cancer Biology of Zhejiang Province, Hangzhou Normal University, Hangzhou, China; ^4^ School of Basic Medicine, Hangzhou Normal University, Hangzhou, China

**Keywords:** lung adenocarcinoma, junctional genes, risk score, prognosis, overall survival

In the published article, there is an error in Figure 4D as published. Kaplan-Meier survival curves based on the JGRS in GSE72094 is incorrectly inserted. After checking the original data, we realized that the error was due to repeated insertion of Kaplan-Meier survival curves of GSE37745 during the figure assembly. In addition, for GSE31210, the red (high JGRS) and blue (low JGRS) lines are labeled oppositely. The corrected Figure 4 appears below.

**FIGURE 4:**
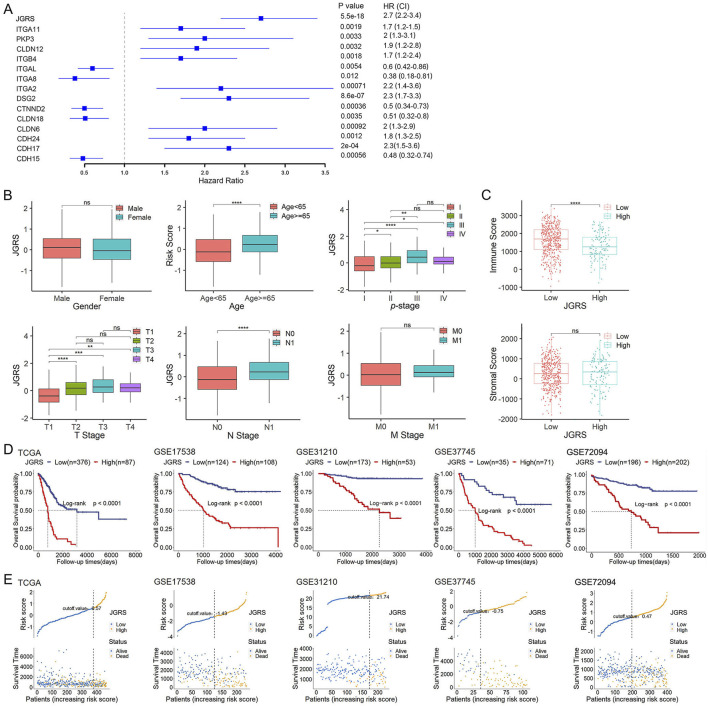
The JGRS can distinguish different clinicopathological features of LUAD. **(A)** A forest plot of the univariate Cox regression analysis of JGRS and 14 genes that were chosen for establishing a prognosis signature. **(B)** Different analyses of JGRS distribution based on sex, age, p-stage, as well as T, N, and M stages in TCGA cohort. **(C)** Distribution of immune and stromal scores between low- and high-JGRS groups in TCGA cohort. **(D)** Kaplan-Meier survival curves based on the JGRS in TCGA and four GEO cohorts. **(E)** JGRS distribution in TCGA and four GEO cohorts.

The authors apologize for this error and state that this does not change the scientific conclusions of the article in any way. The original article has been updated.

