# Supplementary figures and images for: Comprehensive landscape of junctional genes and their association with overall survival of patients with lung adenocarcinoma
Source: Front Mol Biosci. 2024 May 22;11:1380384. doi: 10.3389/fmolb.2024.1380384 (PMC11150628; doi:10.3389/fmolb.2024.1380384)

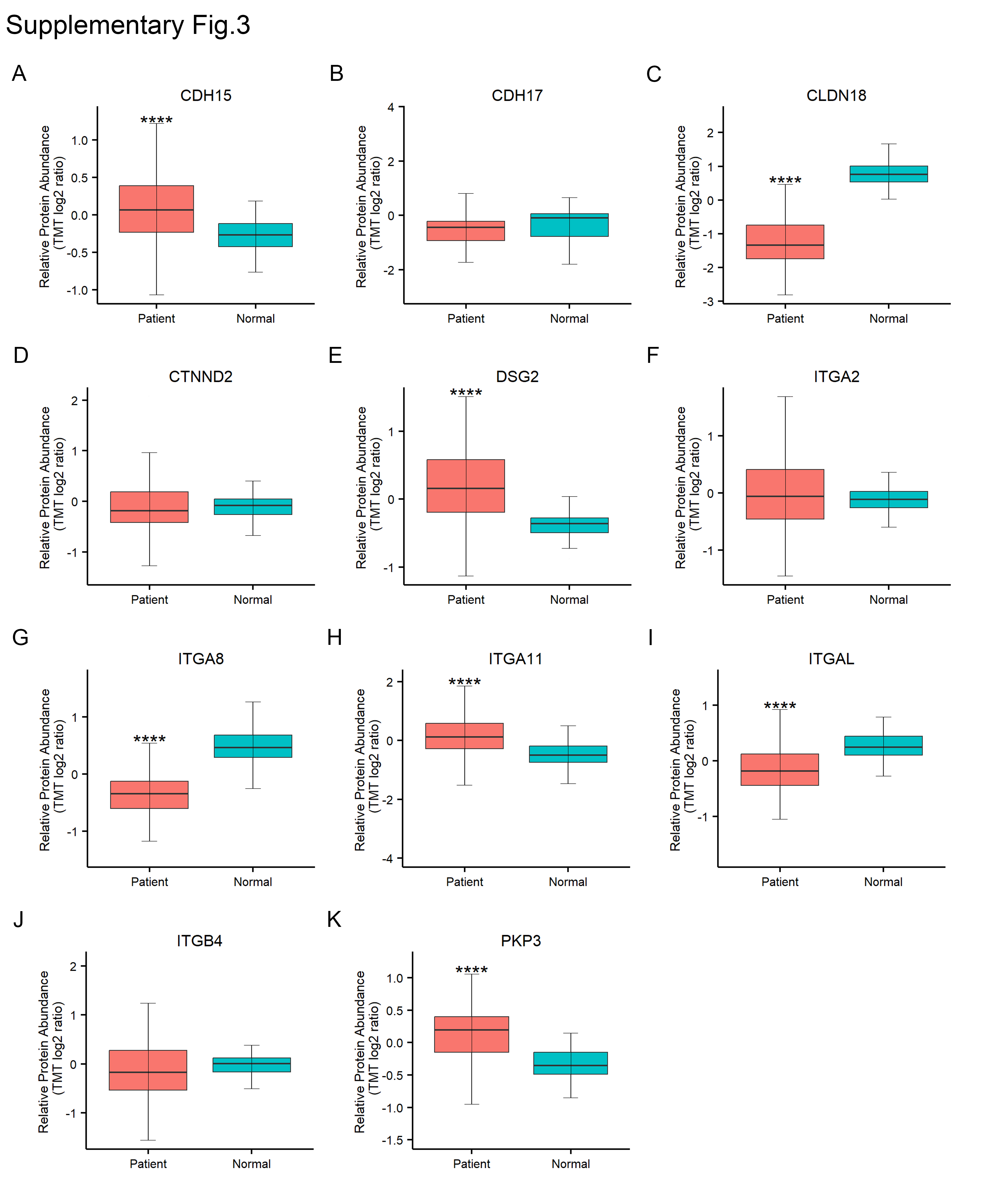

Supplement: Supplementary file 2 [file Image3.jpg]

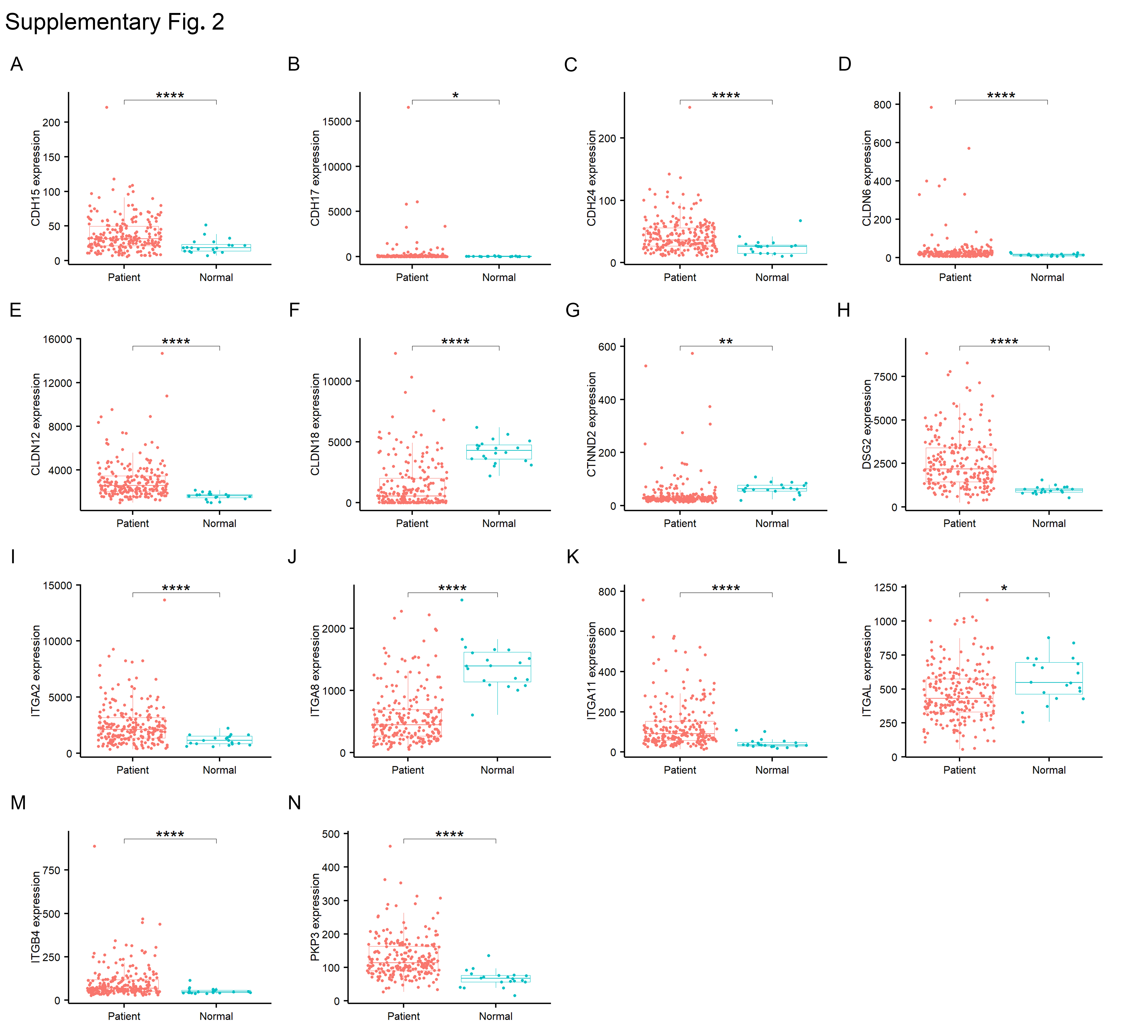

Supplement: Supplementary file 3 [file Image2.jpg]

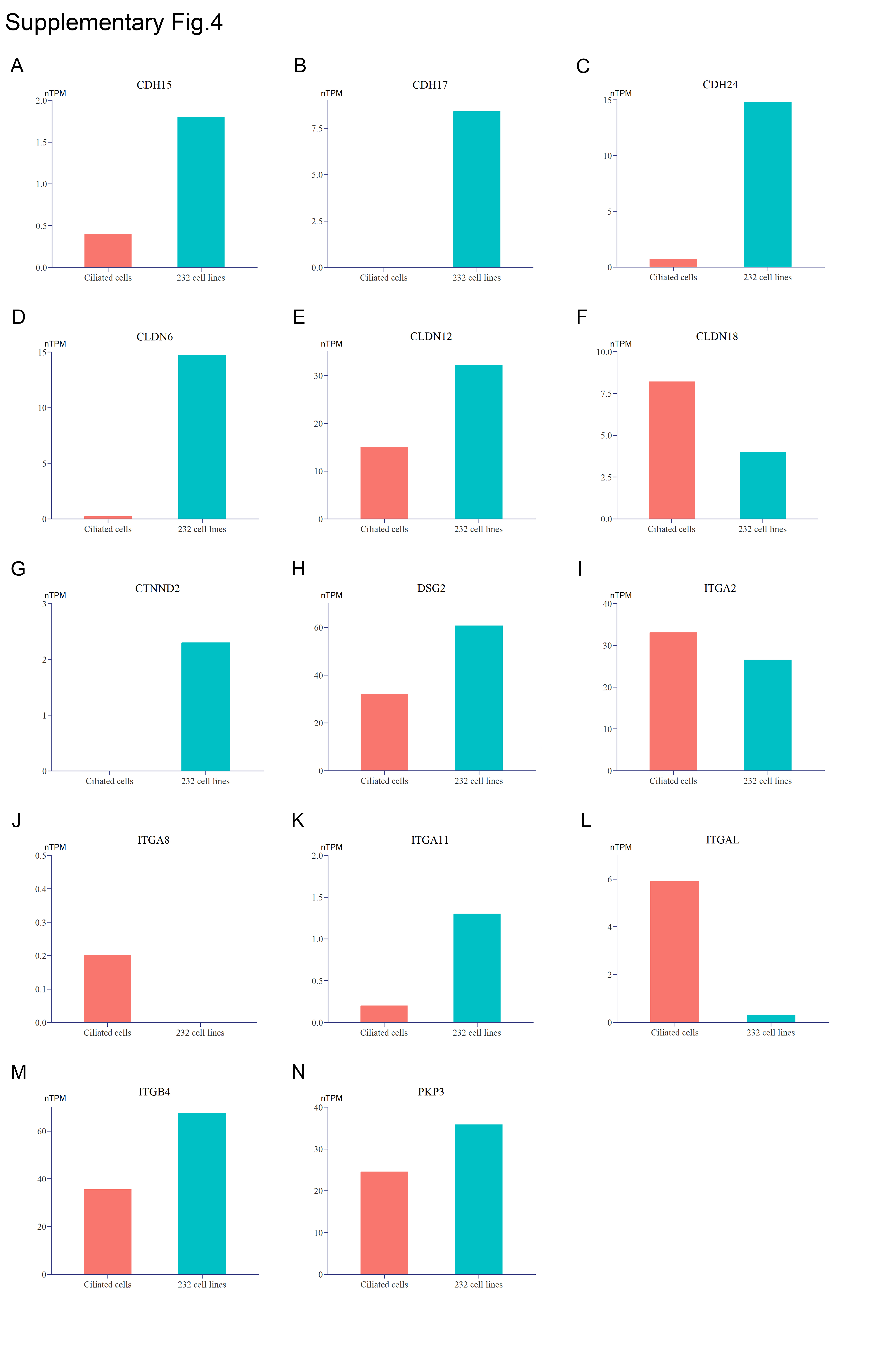

Supplement: Supplementary file 9 [file Image4.jpg]

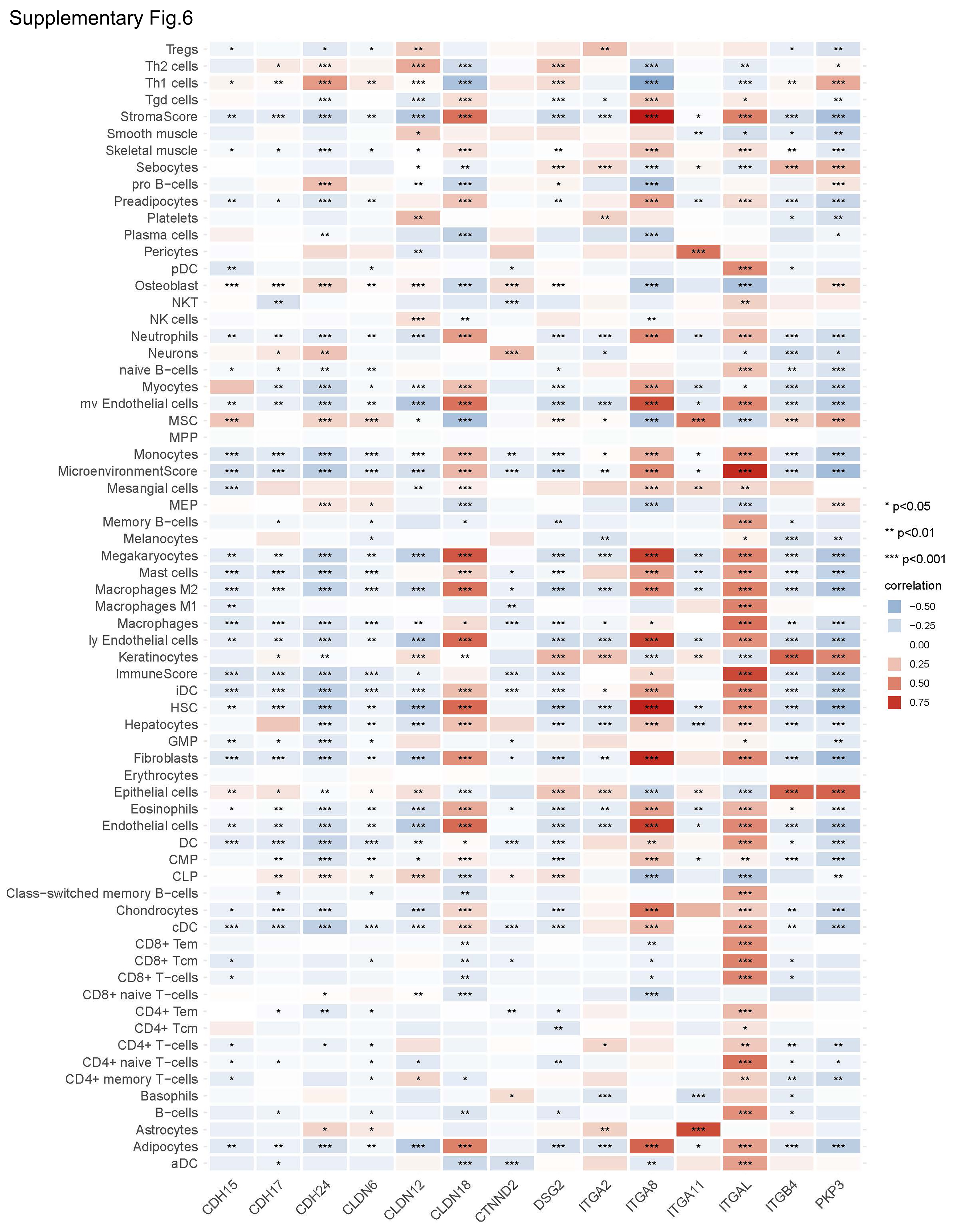

Supplement: Supplementary file 10 [file Image6.JPEG]
