# Supplementary material for: Comprehensive landscape of junctional genes and their association with overall survival of patients with lung adenocarcinoma
Source: Front Mol Biosci. 2024 May 22;11:1380384. doi: 10.3389/fmolb.2024.1380384 (PMC11150628; doi:10.3389/fmolb.2024.1380384)
Supplement: Supplementary file 5 [file Table2.DOCX]

Supplementary Table 2 One hundred and five junctional genes in homo sapiens

| Full name | TCGA gene symbol | Entrez Gene ID |
| --- | --- | --- |
| claudin-1 | CLDN1 | 9076 |
| claudin-2 | CLDN2 | 9075 |
| claudin-3 | CLDN3 | 1365 |
| claudin-4 | CLDN4 | 1364 |
| claudin-5 | CLDN5 | 7122 |
| claudin-6 | CLDN6 | 9074 |
| claudin-7 | CLDN7 | 1366 |
| claudin-8 | CLDN8 | 9073 |
| claudin-9 | CLDN9 | 9080 |
| claudin-10 | CLDN10 | 9071 |
| claudin-12 | CLDN12 | 9069 |
| claudin-14 | CLDN14 | 23562 |
| claudin-15 | CLDN15 | 24146 |
| claudin-16 | CLDN16 | 10686 |
| claudin-17 | CLDN17 | 26285 |
| claudin-18 | CLDN18 | 51208 |
| claudin-19 | CLDN19 | 149461 |
| claudin-20 | CLDN20 | 49861 |
| claudin-22 | CLDN22 | 53842 |
| claudin-23 | CLDN23 | 137075 |
| claudin-24 | CLDN24 | 100132463 |
| claudin-25 | CLDN25 | 644672 |
| claudin-34 | CLDN34 | 100288814 |
| cadherin-1 | CDH1 | 999 |
| cadherin-2 | CDH2 | 1000 |
| cadherin-3 | CDH3 | 1001 |
| cadherin-4 | CDH4 | 1002 |
| cadherin-5 | CDH5 | 1003 |
| cadherin-6 | CDH6 | 1004 |
| cadherin-7 | CDH7 | 1005 |
| cadherin-8 | CDH8 | 1006 |
| cadherin-9 | CDH9 | 1007 |
| cadherin-10 | CDH10 | 1008 |
| cadherin-11 | CDH11 | 1009 |
| cadherin-12 | CDH12 | 1010 |
| cadherin-13 | CDH13 | 1012 |
| cadherin-15 | CDH15 | 1013 |
| cadherin-16 | CDH16 | 1014 |
| cadherin-17 | CDH17 | 1015 |
| cadherin-18 | CDH18 | 1016 |
| cadherin-19 | CDH19 | 28513 |
| cadherin-20 | CDH20 | 28316 |
| cadherin-22 | CDH22 | 64405 |
| cadherin-23 | CDH23 | 64072 |
| cadherin-24 | CDH24 | 64403 |
| cadherin-26 | CDH26 | 60437 |
| α-catenin 1 | CTNNA1 | 1495 |
| α-catenin 2 | CTNNA2 | 1496 |
| α-catenin 3 | CTNNA3 | 29119 |
| β-catenin | CTNNB1 | 1499 |
| p120-catenin(δ-catenin) | CTNND1 | 1500 |
| p120-catenin(δ-catenin) | CTNND2 | 1501 |
| plakoglobin (γ-catenin) | JUP | 3728 |
| vinculin | VCL | 7414 |
| integrin alpha 1 | ITGA1 | 3672 |
| integrin alpha 2 | ITGA2 | 3673 |
| integrin alpha 2B | ITGA2B | 3674 |
| integrin alpha 3 | ITGA3 | 3675 |
| integrin alpha 4 | ITGA4 | 3676 |
| integrin alpha 5 | ITGA5 | 3678 |
| integrin alpha 6 | ITGA6 | 3655 |
| integrin alpha 7 | ITGA7 | 3679 |
| integrin alpha 8 | ITGA8 | 8516 |
| integrin alpha 9 | ITGA9 | 3680 |
| integrin alpha 10 | ITGA10 | 8515 |
| integrin alpha 11 | ITGA11 | 22801 |
| integrin alpha D | ITGAD | 3681 |
| integrin alpha E | ITGAE | 3682 |
| integrin alpha L | ITGAL | 3683 |
| integrin alpha M | ITGAM | 3684 |
| integrin alpha V | ITGAV | 3685 |
| integrin alpha X | ITGAX | 3687 |
| integrin beta 1 | ITGB1 | 3688 |
| integrin beta 2 | ITGB2 | 3689 |
| integrin beta 3 | ITGB3 | 3690 |
| integrin beta 4 | ITGB4 | 3691 |
| integrin beta 5 | ITGB5 | 3693 |
| integrin beta 7 | ITGB7 | 3695 |
| integrin beta 8 | ITGB8 | 3696 |
| talin 2 | TLN2 | 83660 |
| filamin A | FLNA | 2316 |
| filamin B | FLNB | 2317 |
| filamin C | FLNC | 2318 |
| paxillin | PXN | 5829 |
| focal adhesion kinase (FAK) | PTK2 | 5747 |
| desmoglein 1 | DSG1 | 1828 |
| desmoglein 2 | DSG2 | 1829 |
| desmoglein 3 | DSG3 | 1830 |
| desmoglein 4 | DSG4 | 147409 |
| desmocolin 1 | DSC1 | 1823 |
| desmocolin 2 | DSC2 | 1824 |
| desmocolin 3 | DSC3 | 1825 |
| plakophilin 1 | PKP1 | 5317 |
| plakophilin 2 | PKP2 | 5318 |
| plakophilin 3 | PKP3 | 11187 |
| plakophilin 4 | PKP4 | 8502 |
| desmoplakin | DSP | 1832 |
| plectin | PLEC | 5339 |
| dystonin | DST | 667 |
| occludin | OCLN | [100506658](http://www.ncbi.nlm.nih.gov/entrez/query.fcgi?db=gene&cmd=Retrieve&dopt=full_report&list_uids=100506658) |
| tight junction protein 1 | TJP1 | 7082 |
| tight junction protein 2 | TJP2 | 9414 |
| tight junction protein 3 | TJP3 | 27134 |
| junctional adhesion molecule 2 | JAM2 | 58494 |
| junctional adhesion molecule 3 | JAM3 | 83700 |
