# Supplementary material for: Comprehensive landscape of junctional genes and their association with overall survival of patients with lung adenocarcinoma
Source: Front Mol Biosci. 2024 May 22;11:1380384. doi: 10.3389/fmolb.2024.1380384 (PMC11150628; doi:10.3389/fmolb.2024.1380384)
Supplement: Supplementary file 6 [file Table3.DOCX]

Supplementary Table 3 Clinical characteristics of patients in four GEO cohorts

| **Characteristics** | **GSE17538 cohort (N=232)** | | | **GSE31210 cohort (N=226)** | | **GSE37745 cohort (N=106)** | | | **GSE72094 cohort (N=398)** | |
| --- | --- | --- | --- | --- | --- | --- | --- | --- | --- | --- |
|  | **N** | **%** | **N** | | **%** | **N** | **%** | **N** | | **%** |
| Sex |  |  |  | |  |  |  |  | |  |
| Male | 122 | 52.6 | 105 | | 46.5 | 46 | 43.4 | 176 | | 44.2 |
| Female | 110 | 47.4 | 121 | | 53.5 | 60 | 56.6 | 222 | | 55.8 |
| Age |  |  |  | |  |  |  |  | |  |
| Mean (SD) | 64.7 | 13.4 | 59.6 | | 7.4 | 62.9 | 9.22 | 69.4 | | 9.45 |
| Median [Min, Max] | 65.5 | [23.0, 94.0] | 61.0 | | [30.0,76.0] | 64.0 | [40.0,83.0] | 70.0 | | [38.0,89.0] |
| Pathological stage |  |  |  | |  |  |  |  | |  |
| Stage I | 28 | 12.1 | 168 | | 74.3 | 70 | 66.0 | 254 | | 63.8 |
| Stage II | 72 | 31 | 58 | | 25.7 | 19 | 17.9 | 67 | | 16.8 |
| Stage III | 76 | 32.8 | 0 | | 0 | 13 | 12.3 | 57 | | 14.3 |
| Stage IV | 56 | 24.1 | 0 | | 0 | 4 | 3.8 | 15 | | 3.8 |
| Na | 0 | 0 | 0 | | 0 | 0 | 0 | 5 | | 1.3 |
