# Supplementary material for: Comprehensive landscape of junctional genes and their association with overall survival of patients with lung adenocarcinoma
Source: Front Mol Biosci. 2024 May 22;11:1380384. doi: 10.3389/fmolb.2024.1380384 (PMC11150628; doi:10.3389/fmolb.2024.1380384)
Supplement: Supplementary file 8 [file Table4.DOCX]

Supplementary Table 4 The sequences of primers used in real-time PCR

| Genes | Sequences (5’→3’) |
| --- | --- |
| CDH15 | Forward: CGATCAGCGTATCCGAGAACC |
|  | Reverse: TTAGCCTGAAGCGATCAGTCT |
| CDH17 | Forward: AGGCCAAGAACCGAGTCAAAT |
|  | Reverse: GCAACCTGGAGATTGTGAGTAGA |
| CDH24 | Forward: AAGGCGCAATATGTGCTACTG |
|  | Reverse: AAAATGGGTGGATTGTCGTTGA |
| CLDN6 | Forward: TGTTCGGCTTGCTGGTCTAC |
|  | Reverse: CGGGGATTAGCGTCAGGAC |
| CLDN12 | Forward: CTGTGTGGAATCGCCTCAGTA |
|  | Reverse: GTCAGGTTCTTCTCGTTTCTGTT |
| CLDN18 | Forward: ACATGCTGGTGACTAACTTCTG |
|  | Reverse: AAATGTGTACCTGGTCTGAACAG |
| CTNND2 | Forward: GAGCCCCGGCTTAAACACC |
|  | Reverse: CCTGTTCTTTGACTGAGGCGA |
| DSG2 | Forward: CTAACAGGTTACGCTTTGGATGC |
|  | Reverse: GTGAACACTGGTTCGTTGTCAT |
| ITGA2 | Forward: CCTACAATGTTGGTCTCCCAGA |
|  | Reverse: AGTAACCAGTTGCCTTTTGGATT |
| ITGA8 | Forward: TCAGGCGTTCAACCTGGAC |
|  | Reverse: GCGTCGGGTATGTGGAAGTC |
| ITGA11 | Forward: GTGGCAATAAGTGGCTGGTC |
|  | Reverse: GTTCCCGTGGATCACTGGAC |
| ITGAL | Forward: TGCTTATCATCATCACGGATGG |
|  | Reverse: CTCTCCTTGGTCTGAAAATGCT |
| ITGB4 | Forward: GCAGCTTCCAAATCACAGAGG |
|  | Reverse: CCAGATCATCGGACATGGAGTT |
| PKP3 | Forward: GGCCGACTATGACACACTCTC |
|  | Reverse: CTCGGGGAAACCTCAGTGG |
| GAPDH | Forward: GGAGTCAACGGATTTGGT |
|  | Reverse：GTGATGGGATTTCCATTGAT |
